# The mRNA Expression Signature and Prognostic Analysis of Multiple Fatty Acid Metabolic Enzymes in Clear Cell Renal Cell Carcinoma

**DOI:** 10.7150/jca.33024

**Published:** 2019-10-21

**Authors:** Zuohui Zhao, Yueran Liu, Qiang Liu, Fei Wu, Xiaoli Liu, Hongyi Qu, Yijiao Yuan, Juntao Ge, Yue Xu, Hao Wang

**Affiliations:** 1 Department of Pediatric Surgery, The First Affiliated Hospital of Shandong First Medical University, Jingshi Road, No. 16766, Jinan, Shandong 250014, China.; 2 Department of Operatology, School of Medicine, Shandong University, Wenhuaxi Road, No. 44, Jinan, Shandong 250012, China.; 3 Laboratory of Microvascular Medicine, Medical Research Center, The First Affiliated Hospital of Shandong First Medical University, Jingshi Road, No. 16766, Jinan, Shandong 250014, China.; 4 Department of Urology, The First Affiliated Hospital of Shandong First Medical University, Jingshi Road, No. 16766, Jinan, Shandong 250014, China.; 5 Department of Radiation Oncology, Shandong Cancer Hospital Affiliated to Shandong University, Jiyan Road, No. 440, Jinan, Shandong 250117, China.

**Keywords:** clear cell renal cell carcinoma, prognostic analysis, fatty acid synthesis, fatty acid oxidation, overall survival

## Abstract

Renal cell carcinoma (RCC) is a metabolic disease, and accumulating evidences indicate significant alterations in the cellular metabolism, especial aerobic glycolysis and glutamine metabolism, in RCC. However, fatty acid (FA) metabolism has received less attention, and the mRNA expression pattern and prognostic role of FA metabolic enzymes in clear cell RCC (ccRCC) have not been carefully examined. In the current study, we first investigated the mRNA expression profiles of multiple FA metabolic enzymes, i.e., ACLY, ACC, FASN, SCD, CPT1A, HADHA, HADHB, and ACAT1, in 42 ccRCC and 33 normal kidney tissues using the Oncomine database, validated their mRNA expression profiles using GEPIA resource, then evaluated and validated the prognostic significance of these metabolic enzymes in 530 ccRCC patients using Kaplan-Meier plotter and GEPIA analyses respectively. The Oncomine and GEPIA confirmed higher ACLY, SCD, and lower ACAT1 mRNA expression in ccRCC than normal tissues (P<0.05). And further prognostic analysis displayed that overexpression of the some FA anabolic enzymes (FASN) was correlated to poor overall survival (OS), while overexpression of the FA catabolic enzymes (CPT1A, HADHA, HADHB, and ACAT1) was correlated to favorable OS in ccRCC patients. In conclusion, multiple FA metabolic enzymes, such as FASN, HADHA, and ACAT1, were potential prognostic markers of ccRCC, which implied alterations in FA metabolism might be involved in ccRCC tumorigenesis and progression.

## Introduction

Kidney cancer is a common disease worldwide, with an estimated 65,340 newly diagnosed cases and 14,970 cancer-related deaths in the United States in 2018 1. Renal cell carcinoma (RCC), the most common neoplasm in the adult kidney, represents a heterogenous group of tumors. About 80% of RCCs are of the clear-cell type (ccRCC), which is histologically characterized by abundance of lipid droplets (LDs) in the cytoplasm 2, 3. Because RCC is highly aggressive and generally resistant to chemotherapy, the prognosis is poor, especially for advanced RCC. Although recent proteomics and metabolomics analyses have identified numerous potential prognostic biomarkers for RCC 4-6, there is still no widely accepted molecular marker to evaluate the prognosis of patients with RCC yet. And it is urgent to discover potential prognostic markers to monitor tumor progression and molecular therapeutic targets to improve the overall prognosis for advanced RCC patients 7, 8.

**Figure 1:**
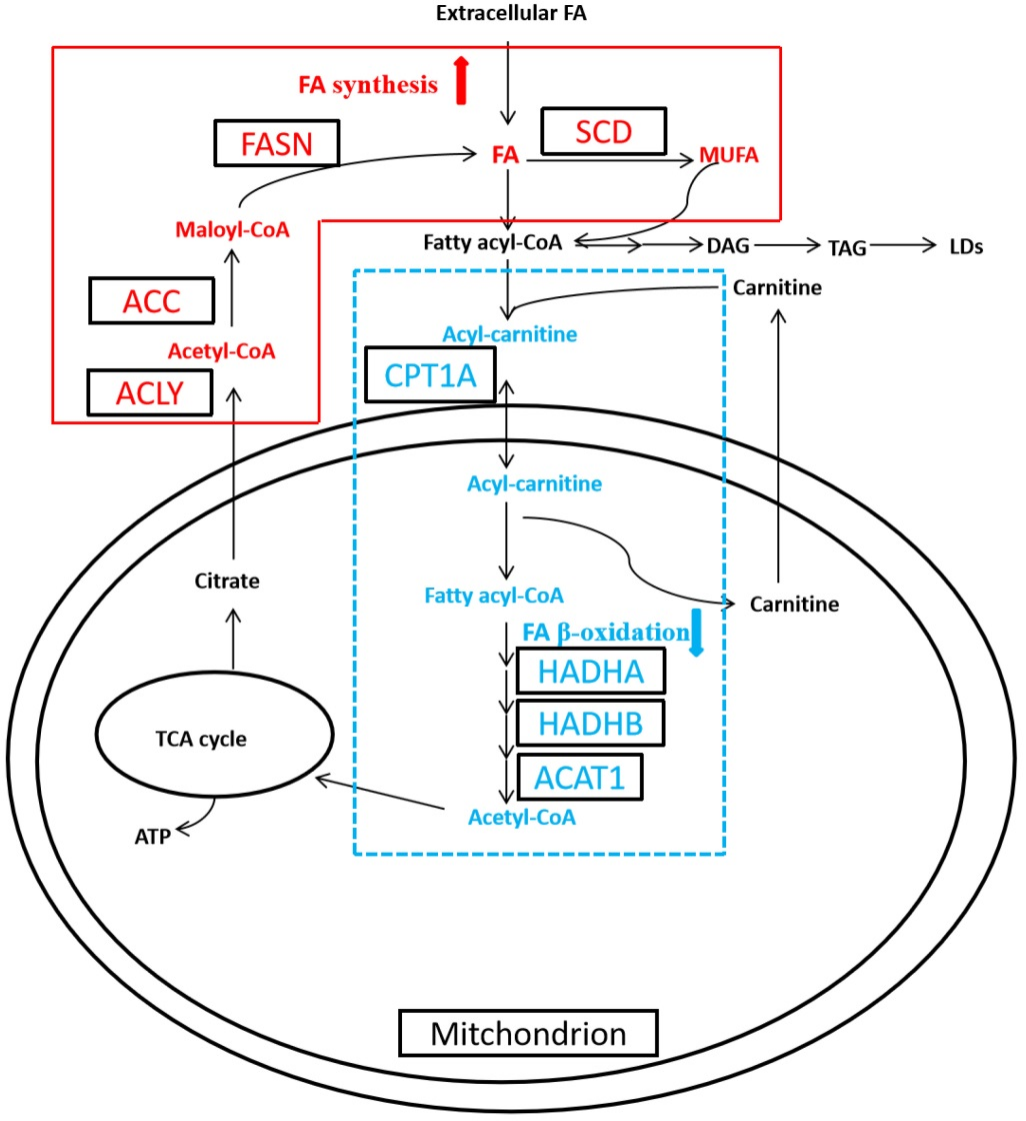
** Altered fatty acid (FA) metabolism in clear cell renal cell carcinoma (ccRCC).** In ccRCC, de novo FA synthesis (FAS) was upregulated, whereas FA β-oxidation (FAO) was downregulated.

Cancer is a disease with complex metabolic perturbations, and RCC manifests significant alterations in the cellular metabolism, such as aerobic glycolysis, glutamine metabolism, and fatty acid (FA) metabolism 9, 10. Thus RCC, characterized by the dysregulation of metabolic pathways, has been recognized as a metabolic disease recently 2, 9. The first well-recognized metabolic change is Warburg effect, which cancer cells shift from mitochondrial oxidative phosphorylation to aerobic glycolysis. And the second metabolic alteration is glutamine metabolism, which increased glutaminolysis generates amount of α‑ketoglutarate to maintain the activity of the mitochondrial tricarboxylic acid (TCA) cycle 11. While alteration in FA metabolism has received less attention, recent studies have started to clarify the relevant role of this metabolic pathway in carcinogenesis 12, 13. FA functions as signaling molecule, storage compound, and structural component of the cell membrane, all of which are essential for cancer cell growth and progression. FA is regulated by their synthesis (FA synthesis, FAS) and catabolism (FA oxidation, FAO; also known as β-oxidation) 9, 13. De novo synthesis of FA is activated in cancer cell, which is catalyzed by ATP citrate lyase (ACLY), acetyl-CoA carboxylase (ACC) and fatty acid synthase (FASN), then converted to monounsaturated FA (MUFA) to generate triacylglycerol (TAG), the composition of LDs, by stearoyl-CoA desaturase (SCD) (Figure 1). Meanwhile FA β-oxidation (FAO) remains obscure in cancer cell, which is catalyzed by carnitine palmitoyltransferase 1A (CPT1A), hydroxyl-coenzyme A dehydrogenase alpha subunit (HADHA), hydroxyl-coenzyme A dehydrogenase beta subunit (HADHB), and acetyl-coenzyme A acetyltransferase 1 (ACAT1) 9, 14.

In addition to DNA mutation and protein expression, mRNA expression patterns were also commonly used to predict the outcomes of cancer patients 15-17. Though multiple mRNA biomarkers have been reported in cancers, few results have been replicated in independent cohorts because of different laboratory settings, cancer grades and/or stages 15. Thanks to the advances in high-throughput technology, especially microarrays and next-generation sequencing, the public online biomedical databases have increased exponentially, which facilitates to decipher the complex molecular pattern in cancer 15, 16. Recently, we and others have identified some dysregulated metabolic pathways, such as glycolysis, FAO, and tryptophan degradation in RCC by proteomic approaches 4, 5. Further research found that HADHA, a downregulated FA metabolic enzyme, was recognized as a favorable prognostic marker for ccRCC patients 7. In this current study, we investigated the mRNA expression profiles of multiple FA metabolic enzymes by Oncomine analysis, validated their differential expression by independent GEPIA (Gene Exoression Profiling Interactive Analysis) analysis, then evaluated their prognostic significances of these metabolic enzymes by Kaplan-Meier plotter and GEPIA analyses, which aimed to discover potential prognostic markers or therapeutic targets in ccRCC, and tried to clarify the FA metabolic abnormalities underlying ccRCC carcinogenesis.

## Materials and Methods

### Oncomine database analysis

The Oncomine database (https://www.oncomine.org) based on existing cancer microarray data sets was used to analyze the mRNA expression status of the multiple FA metabolic enzymes in ccRCC as described previously 8. Oncomine data set filters were set as data type (mRNA), and the search pattern was set as gene, including ACLY, ACC (gene ACACA), FASN, SCD, CPT1A, HADHA, HADHB, and ACAT1. Then the differential expression signature of their mRNA between ccRCC and normal tissues was displayed, which included sample size, *P* value, fold change, and statistical box plot.

### GEPIA database analysis

The GEPIA database (http://gepia.cancer-pku.cn/) was used to validate the expression and prognosis of the FA metabolic enzymes in ccRCC patients 18. In the web-based resource, all the eight genes of FA metabolic enzymes were loaded into the server, and the data from the Cancer Genome Atlas (TCGA) and Genotype-Tissue Expression (GTEx) were available for validation analysis. To be specific, 523 ccRCC and 100 normal kidney specimens were used for confirmation their differential expression, and 516 ccRCC samples were used for validation their overall survival (OS). The hazard ratio (HR) and Log rank *P* value were calculated automatically on the webpage.

### RT-PCR analysis

To validate the differential expression of ACAT1 mRNA, reverse-transcription PCR (RT-PCR) was performed according to the manufacture's instruction. The Primers for ACAT1: forward, 5'-GGAGGTGAAGGACAAGCTCC-3', reverse, 5'-TCTACAGCAGCGTCAGCAAA-3', and for GAPDH: forward, 5'-TTCCAGCCTTCCTTCCTGG-3', reverse, 5'-TTGCGCTCAGGAGGAGCAAT-3'.

### Kaplan-Meier plotter database analysis

The Kaplan-Meier plotter database (www.kmplot.com) was used to analyze the correlation between mRNA level of each FA metabolic enzymes and prognosis of ccRCC patients as described previously 16, 19. In the public online database, the eight FA metabolic enzymes genes (ACLY, ACACA, FASN, SCD, CPT1A, HADHA, HADHB, and ACAT1), were loaded into the database and accessed their prognosis (OS) by the Kaplan-Meier survival plot (n=530). The HR with 95% confidence intervals (CI) and Log rank *P* value were calculated automatically on the webpage. The relationship between the gene expression and clinicopathological parameters, i.e., gender and clinical stages, of ccRCC, was also investigated.

### Statistical analysis

The SPSS 21.0 software (SPSS Inc, Chicago, IL) was used for statistical analysis. For Oncomine and RT-PCR analyses, the mRNA expression values of the metabolic enzymes between ccRCC and normal tissues were analyzed using Student's t-test. For GEPIA validation analysis, their expression levels were calculated using ANOVA analysis, and the correlation between the mRNA expression of the enzymes and OS rate was calculated by the Kaplan-Meier curve and log-rank test automatically. For Kaplan-Meier plotter analysis, the correlation between their mRNA expression and OS was also calculated by the log-rank test automatically. *P* values less than 0.05 were considered statistically significant.

## Results

### The mRNA expression profile of the FA metabolic enzymes in ccRCC

**Figure 2:**
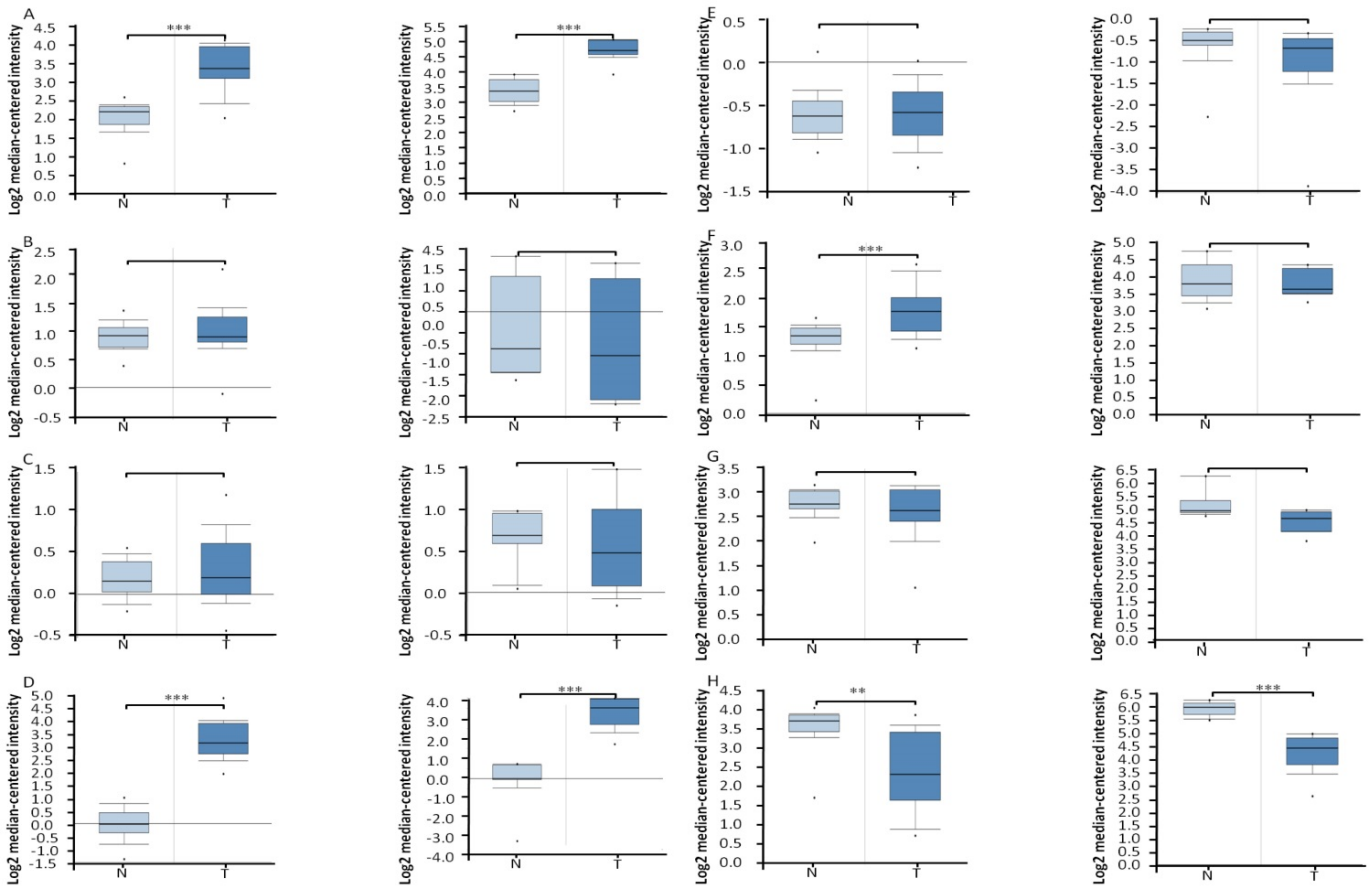
** The box plots demonstrated the mRNA expression profile of FA metabolic enzymes in ccRCC and in normal kidney tissues, which was downloaded from Oncomine database.** The box plots showed the relative mRNA expression of ACLY (A), ACC (B), FASN (C), SCD (D), CPT1A(E), HADHA (F), HADHB (G), and ACAT1 (H), in U133A (left, 32 cases of ccRCC and 23 cases of normal kidney tissues) and U133A/B (right, 10 cases of ccRCC and 10 cases of normal kidney tissues) microarrays datasets, repectively. * P<0.05, ** P<0.01, *** P<0.001.

First, Oncomine database was utilized to analyze the transcriptional profiling of the FA metabolic enzymes from the existing microarray data sets. Data of Affymetrix U133A microarray (32 ccRCCs and 23 normal tissues) 20 and Affymetrix U133A/B microarray (10 ccRCCs and 10 normal kidney tissues) 21 from the publicly available Oncomine database were utilized to examine the mRNA expression level of multiple FA metabolic enzymes in ccRCC. First, we compared their mRNA expression of four enzymes involved in FA biosynthesis, ACLY, ACC, FASN and SCD. As illustrated in Figure 2, the mean ACLY mRNA expression was elevated 2.49-fold (*P*<0.001, U133A microarray) and 2.53-fold (*P*<0.001, U133A/B microarray) in ccRCC compared with normal kidney tissues, respectively. Similar data also displayed for SCD, which were increased to 9.77-fold and 10.15-fold in ccRCC as compared with normal tissues, and the difference was statistical significance (*P*<0.001, respectively). Interestingly, the mRNA levels of another two FA anabolic enzymes, ACC and FASN, were stable, and the difference was not statistical significance. To be specific, there were a slightly increasing trend in U133A microarray, 1.08-fold (*P*=0.139 for ACC) and 1.09-fold (*P*=0.109 for FASN), while a slightly decreasing trend in U133A/B microarray, -1.21-fold (*P*=0.689 for ACC), and -1.08-fold (*P*=0.719 for FASN).

The mRNA levels of four enzymes involved in FAO, ACAT1, CPT1A, HADHA and HADHB, were also analyzed by Oncomine database (Figure 2). The ACAT1 mRNA level was down-regulated 2.26-fold (*P*=0.001, U133A microarray) and 3.23-fold (*P*<0.001, U133A/B microarray) in ccRCC compared with normal kidney tissues, respectively, which was consistent with its protein expression by our immunoblotting analysis 4. The HADHA mRNA level was increased to 1.38-fold (*P*<0.001) in U133A microarray, while it was stable (-1.042-fold, *P*=0.619) in U133A/B microarray, and further research with more samples were needed to clarify this inconsistency. The mRNA levels of another two FA catabolic enzymes, CPT1A and HADHB, were stable both in U133A microarray (1.02-fold, *P*=0.402 for CPT1A, -1.16-fold, *P*=0.948 for HADHB, respectively) and U133A/B microarray (-1.32-fold, *P*=0.843 for CPT1A, -1.50-fold, *P*=0.997 for HADHB, respectively).

For mRNA expression analysis, the higher expression levels of ACLY and SCD (*P*<0.05), and the lower expression level of ACAT1 (*P*<0.05), were all independently validated using alternative GEPIA analysis (Figure S1). And further RT-PCR analysis also confirmed the lower expression of ACAT1 mRNA in ccRCC tissues (*P*=0.002, Figure S2).

### The prognostic analysis of FA metabolic enzymes in ccRCC

**Figure 3:**
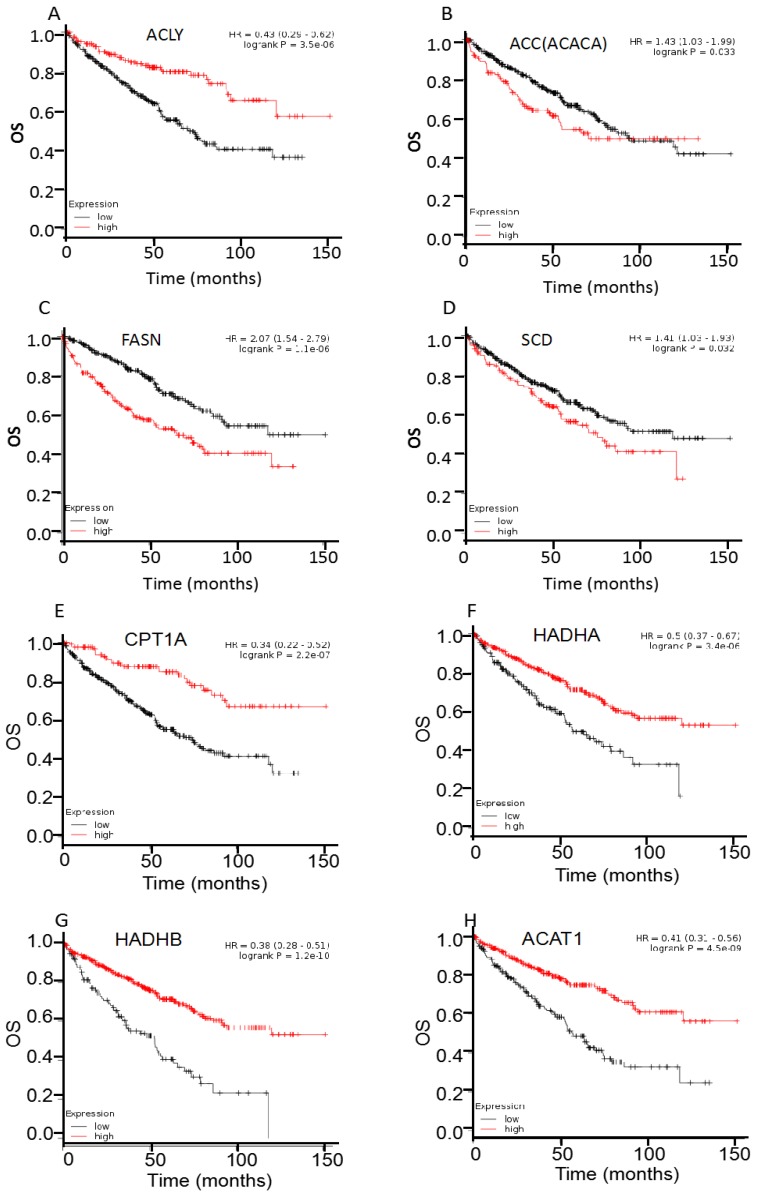
Overall survival (OS) curves of ACLY (A), ACC (B), FASN (C), SCD (D), CPT1A(E), HADHA (F), HADHB (G), and ACAT1 (H), were plotted for 530 ccRCC patients.

For survival analysis, public online resource Kaplan-Meier plotter was utilized to analyze the clinical significance of individual genes on OS of 530 cases ccRCC (Figure 3, table 1). First, the prognostic significance of four enzymes involved in FAS was examined in ccRCC patients. Results showed that most of them were hazard factors, i.e., overexpression of ACC (HR 95%CI =1.43 (1.03-1.99), *P*=0.033), FASN (HR 95%CI =2.07 (1.54-2.79), *P*<0.001), and SCD (HR 95%CI =1.41 (1.03-1.93), *P*=0.032) exhibited a poor OS in ccRCC patients. Whereas ACLY was a favorable factor, i.e., the HR was 0.43 (95%CI: 0.29-0.62, *P*<0.001). Then the four enzymes involved in FAO were analyzed, and all of them were protective factors, i.e., overexpression of CPT1A (HR 95%CI =0.34 (0.22-0.52), *P*<0.001), HADHA (HR 95%CI =0.50 (0.37-0.67), *P*<0.001), HADHB (HR 95%CI =0.38 (0.28-0.51), *P*<0.001), and ACAT1 (HR 95%CI =0.41 (0.31-0.56), *P*<0.001) showed a favorable OS in ccRCC patients. Moreover, the survival analysis of HADHA mRNA was consistent with our previous reports of its protein expression 7.

On the basis of the OS analysis of these enzymes, we further investigated their prognostic roles in different clinicopathological parameters (i.e., clinical stages, gender) of ccRCC patients. Table 2 and table 3 presented the correlation of their mRNA expression with OS in different clinical stages of ccRCC patients, respectively. For FA anabolic enzymes (table 2), increased expression of FASN was correlated with unfavorable OS in stage II, III, and IV ccRCC patients (HR 95% CI=3.33 (1.10-10.09), 2.25 (1.27-3.97), and 2.74 (1.66-4.53), respectively), while there was no difference in stage I ccRCC patients (*P*>0.05). Similar results were seen for ACC, that overexpression of ACC was correlated with adverse OS in stage IV (HR 95% CI=2.36 (1.42-3.91)) ccRCC patients. However, enhanced ACLY was correlated with better OS in stage I, II, and III ccRCC patients (HR 95% CI=0.39 (0.19-0.82), 0.16 (0.03-0.72), and 0.50 (0.29-0.89), respectively), while there was no difference in stage Ⅳ ccRCC patients (*P*>0.05). As for FA catabolic enzymes (table 3), in stage I, III, and IV ccRCC patients, we found that CPT1A (HR 95% CI=0.35 (0.17-0.72), 0.54 (0.30-0.96), 0.49 (0.28-0.87), respectively), ACAT1 (HR 95% CI=0.50 (0.28-0.91), 0.52 (0.30-0.92), 0.45 (0.28-0.74), respectively) mRNA expressions were associated with favorable OS. And in stage II, III, and IV ccRCC patients, HADHB (HR 95% CI=0.31 (0.10-0.98), 0.47 (0.27-0.84), 0.42 (0.26-0.69), respectively) mRNA expressions was associated with favorable prognosis. Meanwhile, HADHA mRNA was related to better OS in stage I (HR 95% CI=0.44 (0.24-0.82)) and IV (HR 95% CI=0.37 (0.21-0.61)) ccRCC patients.

With regard to gender, the prognostic values of individual enzymes were further investigated in ccRCC patients. As illustrated in table 4, FASN overexpression was correlated with poor OS in both female (HR 95% CI=2.42 (1.47-4.00)) and male (HR 95% CI=2.20 (1.51-3.19)), and ACC was only correlated with poor OS in male (HR 95% CI=1.77 (1.19-2.63)), while ACLY was correlated with favorable OS in both female (HR 95% CI=0.28 (0.15-0.52)) and male (HR 95% CI=0.52 (0.35-0.77)). Moreover, the four enhanced catabolic enzymes, including CPT1A, HADHA, HADHB, and ACAT1, were all correlated with better OS in both male and female ccRCC patients (*P*<0.05).

For GEPIA validation analysis, most of the FA metabolic enzymes, including ACLY, FASN, CPT1A, HADHA, HADHB, and ACAT1, showed the consistent prognosis (OS) with the same follow-up period (150 months) in another 516 ccRCC patients (Figure S3). Specifically, overexpression of FASN (HR=2.10, *P*<0.001) exhibited a poor OS, and overexpression of ACLY (HR=0.52, *P*<0.001), CPT1A (HR=0.47, *P*<0.001), HADHA (HR=0.48, *P*<0.001), HADHB (HR=0.38, *P*<0.001), and ACAT1 (HR=0.42, *P*<0.001) showed a favorable OS in ccRCC patients. Interestingly, the two enzymes involved in FAS, i.e., ACACA and SCD, were not recognized as hazard factors by GEPIA analysis. To be specific, the* P* values were 0.19 for ACACA (HR=1.20), and 0.57 for SCD (HR=0.92).

## Discussion

Metabolic reprogramming, or altered metabolism, is the hallmark of cancer, and enables cancer cell to survive and proliferate even under deleterious conditions 9. In addition to the well-known aerobic glycolysis and glutamine metabolism, recent researches has started to reveal FA metabolic reprogramming in carcinogenesis, which facilitates to decipher the complex molecular pattern in tumorigenesis and discover potential prognostic markers for clinical intervene of RCC 2, 9, 10, 13, 14. Cancer cells prefer to de novo FA synthesis (FAS) instead of uptake exogenous FA for rapid proliferation 13. The de novo FAS pathway converts citrate to acetyl-coenzyme A (CoA), malonyl-CoA, eventually, bioactive FAs through multiple enzymatic reactions catalyzed by ACLY, ACC and FASN (Figure 1). Then FA is elongated and desaturated by SCD to generate MUFA as well as TAG, which was stored in the LDs as the energy source. During the catabolic process, fatty acyl-CoA, the activated FA, is converted to fatty acyl-carnitine and translocated to mitochondria via CPT1A. Then, fatty acyl-carnitine undergoes FAO pathway, which was catalyzed by HADHA, HADHB, and ACAT1, followed by the TCA cycle 9. De novo FAS is activated in most of cancer, while FAO is largely unknown during cancer pathogenesis 2, 13, 14.

In the current study, we first systematically compared the mRNA expression profile of the eight FA metabolic enzymes between ccRCC and normal tissues using Oncomine database, validated their differential expression using GEPIA and RT-PCR analyses, then comprehensively investigated the prognostic significance of these enzymes in ccRCC patients using the Kaplan-Meier plotter and GEPIA analyses. We found the mRNA expression levels of ACLY and SCD were higher, while ACAT1 was lower, in ccRCC comparing with normal tissues (*P*<0.05) using independent verification and validation analyses. And further Kaplan-Meier plotter prognostic analysis displayed that overexpression of the major FA anabolic enzymes (FASN, ACC, and SCD) were correlated to poor OS, while overexpression of the FA catabolic enzymes (CPT1A, HADHA, HADHB, and ACAT1) were correlated to favorable OS in ccRCC patients (*P*<0.05). Additionally, detailed clinical stage and gender analyses also confirmed their OS in ccRCC. Then, the prognostic significance of most of the FA metabolic enzymes (FASN, CPT1A, HADHA, HADHB, and ACAT1) was validated using independent GEPIA analyses (*P*<0.05). To the best of our knowledge, this is the first report about the mRNA expression profile and prognostic role of multiple FA metabolic enzymes, such as ACLY, HADHA, and ACAT1 in ccRCC simultaneously, which indicated they were potential prognostic markers of ccRCC, and FA metabolism abnormality might be involved in ccRCC tumorigenesis and progression.

De novo FAS is activated in RCC, and increasing literatures indicate that enhanced FA anabolic enzymes are potential prognostic markers or therapeutic targets of ccRCC 9. ACC converts acetyl-CoA to malonyl-CoA as the first rate-limiting step in de novo lipogenesis. RNA signature analysis showed that ACC mRNA was increased in ccRCC, and further prognosis analysis displayed increased ACC protein was a poor survival for ccRCC 17, which was consistent with our result. FASN is the central enzyme in de novo lipogenesis, and SCD generates MUFA for LDs formation. In agreement with our finding, the mRNA and protein levels of both FASN and SCD were reportedly increased in ccRCC, and their overexpressions were correlated with poor patient survival 9, 15, 17, 22, 23. As for ACLY, a critical enzyme that connects glucose metabolism to liposynthesis, its mRNA expression was significantly higher in ccRCC tissues compared with normal tissues 24, further analysis showed it was a favorable survival marker for ccRCC 17, which was consistent with our result. Previous reports also demonstrated all of the FAS enzymes were potential therapeutic targets for pharmacological intervention of ccRCC 9.

FAO is vital for cancer cell to grow and survive, recent studies showed that FAO pathway was inhibited in ccRCC 13. Multiple proteomics analyses have demonstrated that enzymes involved in FAO were inhibited in ccRCC tissues 4, 5. CPT1A is the rate-limiting enzyme of mitochondrial β-oxidation, and it has been recognized as a potential therapeutic target for hepatocellular carcinoma (HCC) and ccRCC 3, 14. Du et al. also reported that CPT1A expression was decreased in ccRCC, and its lower expression was associated with poor patient outcome 3. ACAT1 catalyzes the formation of acetoacetyl-CoA from two acetyl-CoA molecules, and proteomics analysis suggested that ACAT1 was downregulated in ccRCC 4, 6. Further survival analysis indicated it was a favorable marker for ccRCC, which was the first time to report it as a potential prognostic marker in ccRCC. Mitochondrial trifunctional protein (TFP), composed of four α (HADHA) and four β (HADHB) subunits, catalyzes the last three steps of mitochondrial β-oxidation of long chain FAs 7. Whereas no consistent result was concluded for HADHA mRNA expression in ccRCC, further survival analysis displayed it was a better prognostic factor for ccRCC, which was consistent with our previous result 7. HADHB was downregulated in colorectal cancer (CRC), and further functional analysis indicated that it reduced cancer cell migration and invasiveness 25. HADHB expression and prognostic significance in ccRCC have not been reported previously.

## Conclusion

In conclusion, we elucidated the mRNA expression dysregulation of multiple FA metabolic enzymes, i.e., ACLY, ACC, FASN, SCD, CPT1A, HADHA, HADHB, and ACAT1, in ccRCC and normal tissues using Oncomine and GEPIA analyses, then evaluated the prognostic significance of these metabolic enzymes in ccRCC patients using Kaplan-Meier plotter and GEPIA analyses. This was the first time to demonstrate the mRNA expression profile and prognostic role of multiple FA metabolic enzymes in ccRCC simultaneously, which concluded FA metabolic enzymes, such as ACLY, HADHA, and ACAT1, were potential prognostic markers of ccRCC. In addition to glucose and glutamine, cancer cell is also highly dependent on FA for survival, and FA metabolism reprogramming might be involved in ccRCC tumorigenesis and progression.

## Supplementary Material

Supplementary figures.

## Figures and Tables

**Table 1:** Correlation of multiple enzymes mRNA expression with overall survival in ccRCC patients (n=530)

Enzyme	Gene	HR (95% CI)	P value
ACLY	ACLY	0.43 (0.29-0.62)	<0.001***
ACC	ACACA	1.43 (1.03-1.99)	0.033*
FASN	FASN	2.07 (1.54-2.79)	<0.001***
SCD	SCD	1.41 (1.03-1.93)	0.032*
CPT1A	CPT1A	0.34 (0.22-0.52)	<0.001***
HADHA	HADHA	0.50 (0.37-0.67)	<0.001***
HADHB	HADHB	0.38 (0.28-0.51)	<0.001***
ACAT1	ACAT1	0.41 (0.31-0.56)	<0.001***

Note: * P<0.05, ** P<0.01, *** P<0.001.

**Table 2:** Correlation of multiple anabolic enzymes mRNA expression with overall survival in different clinical stage of ccRCC patients (n=530)

Enzyme	Stage	n^ 1^	HR (95% CI)	P value ^2^
ACLY	I	265	0.39 (0.19-0.82)	0.010*
	II	57	0.16 (0.03-0.72)	0.007 **
	III	123	0.50 (0.29-0.89)	0.016*
	IV	82	0.72 (0.42-1.23)	0.226
ACC	I	265	0.65 (0.31-1.36)	0.249
	II	57	0.64 (0.21-1.97)	0.438
	III	123	1.33 (0.75-2.33)	0.325
	IV	82	2.36 (1.42-3.91)	<0.001***
FASN	I	265	0.64 (0.35-1.16)	0.135
	II	57	3.33 (1.10-10.09)	0.025*
	III	123	2.25 (1.27-3.97)	0.004 **
	IV	82	2.74 (1.66-4.53)	<0.001***
SCD	I	265	0.81 (0.42-1.56)	0.531
	II	57	not applicable ^3^	- **^#^**
	III	123	0.68 (0.39-1.20)	0.179
	IV	82	1.28 (0.77-2.14)	0.338

Note: 1. The total number was 527, because there were missing expression values and/ or incomplete survival data, 2. * P<0.05, ** P<0.01, *** P<0.001, 3. # The detailed 95% CI was 0-infinitive, P value was not analyzed.

**Table 3:** Correlation of multiple catabolic enzymes mRNA expression with overall survival in different clinical stage of ccRCC patients (n=530)

Enzyme	Stage	n^ 1^	HR (95% CI)	P value ^2^
CPT1A	I	265	0.35 (0.17-0.72)	0.003**
	II	57	0.30 (0.07-1.37)	0.100
	III	123	0.54 (0.30-0.96)	0.032*
	IV	82	0.49 (0.28-0.87)	0.013*
HADHA	I	265	0.44 (0.24-0.82)	0.008**
	II	57	not applicable ^3^	- **^#^**
	III	123	0.58 (0.32-1.05)	0.066
	IV	82	0.37 (0.21-0.61)	<0.001***
HADHB	I	265	0.54 (0.28-1.05)	0.064
	II	57	0.31 (0.10-0.98)	0.036*
	III	123	0.47 (0.27-0.84)	0.008**
	IV	82	0.42 (0.26-0.69)	<0.001***
ACAT1	I	265	0.50 (0.28-0.91)	0.020*
	II	57	0.42 (0.14-1.30)	0.122
	III	123	0.52 (0.30-0.92)	0.022*
	IV	82	0.45 (0.28-0.74)	0.001**

Note: 1. The total number was 527, because there were missing expression values and/ or incomplete survival data, 2. * P<0.05, ** P<0.01, *** P<0.001, 3. # The detailed 95% CI was 0-infinitive, P value was not analyzed.

**Table 4:** Correlation of multiple catabolic enzymes mRNA expression with overall survival in different gender of ccRCC patients (n=530)

Enzyme	Stage	n	HR (95% CI)	P value
ACLY	female	186	0.28 (0.15-0.52)	<0.001***
	male	344	0.52 (0.35-0.77)	0. 001**
ACC	female	186	0.68 (0.41-1.12)	0.131
	male	344	1.77 (1.19-2.63)	0.004**
FASN	female	186	2.42 (1.47-4.00)	<0.001***
	male	344	2.20 (1.51-3.19)	<0.001***
SCD	female	186	0.71 (0.42-1.21)	0.211
	male	344	1.40 (0.97-2.04)	0.074
CPT1A	female	186	0.30 (0.18-0.49)	<0.001***
	male	344	0.55 (0.38-0.81)	0.002**
HDHA	female	186	0.34 (0.20-0.57)	0.008**
	male	344	0.51 (0.35-0.77)	<0.001***
HDHB	female	186	0.35 (0.21-0.59)	<0.001***
	male	344	0.40 (0.27-0.58)	<0.001***
ACAT1	female	186	0.27 (0.16-0.46)	<0.001***
	male	344	0.39 (0.27-0.57)	<0.001***

Note: * P<0.05, ** P<0.01, *** P<0.001.

## Abbreviations

RCC: renal cell carcinoma
ccRCC: clear cell renal cell carcinoma
FA: fatty acid
FAS: fatty acid synthesis
FAO: fatty acid oxidation
OS: overall survival
TCA: tricarboxylic acid
ACLY: ATP citrate lyase
ACC: acetyl-CoA carboxylase
FASN: fatty acid synthase
TAG: triacylglycerol
LD: lipid droplet
SCD: stearoyl-CoA desaturase
CPT1A: carnitine palmitoyltransferase 1A
HADHA: hydroxyl-coenzyme A dehydrogenase alpha subunit
HADHB: hydroxyl-coenzyme A dehydrogenase beta subunit
ACAT1: acetyl-coenzyme A acetyltransferase 1
HR: hazard ratio
CI: confidence intervals
