# Supplementary material for: The mRNA Expression Signature and Prognostic Analysis of Multiple Fatty Acid Metabolic Enzymes in Clear Cell Renal Cell Carcinoma
Source: J Cancer. 2019 Oct 21;10(26):6599–607. doi: 10.7150/jca.33024 (PMC6856888; doi:10.7150/jca.33024)
Supplement: Supplementary file 1 — Supplementary figures. [file jcav10p6599s1.pdf]

Supplementary materials

The mRNA Expression Signature and Prognostic Analysis of Multiple Fatty Acid Metabolic Enzymes in Clear Cell Renal Cell Carcinoma

Zuohui Zhao[1 *], Yueran Liu[2], Qiang Liu[3], Fei Wu[4], Xiaoli Liu[5], Hongyi Qu[1], Yijiao Yuan[1], Juntao Ge[1], Yue Xu[1], Hao Wang[1]

The affiliation(s) and address(es):

[1] Department of Pediatric Surgery, The First Affiliated Hospital of Shandong First Medical University, Jingshi Road, No. 16766, Jinan, Shandong 250014, China

[2] Department of Operatology, School of Medicine, Shandong University, Wenhuaxi Road, No. 44, Jinan, Shandong 250012, China

[3] Laboratory of Microvascular Medicine, Medical Research Center, The First Affiliated Hospital of Shandong First Medical University, Jingshi Road, No. 16766, Jinan, Shandong 250014, China

[4] Department of Urology, The First Affiliated Hospital of Shandong First Medical University, Jingshi Road, No. 16766, Jinan, Shandong 250014, China

[5] Department of Radiation Oncology, Shandong Cancer Hospital Affiliated to Shandong University, Jiyan Road, No. 440, Jinan, Shandong 250117, China

***Corresponding author:** Zuohui Zhao, Ph.D., Department of Pediatric Surgery, The First Affiliated Hospital of Shandong First Medical University, Jingshi Road, No. 16766, Jinan, Shandong 250014, China. Tel: 086-531-89269610, Email: zhaozuohui@126.com.

Running title: The expression and prognosis of FA metabolic enzymes in ccRCC

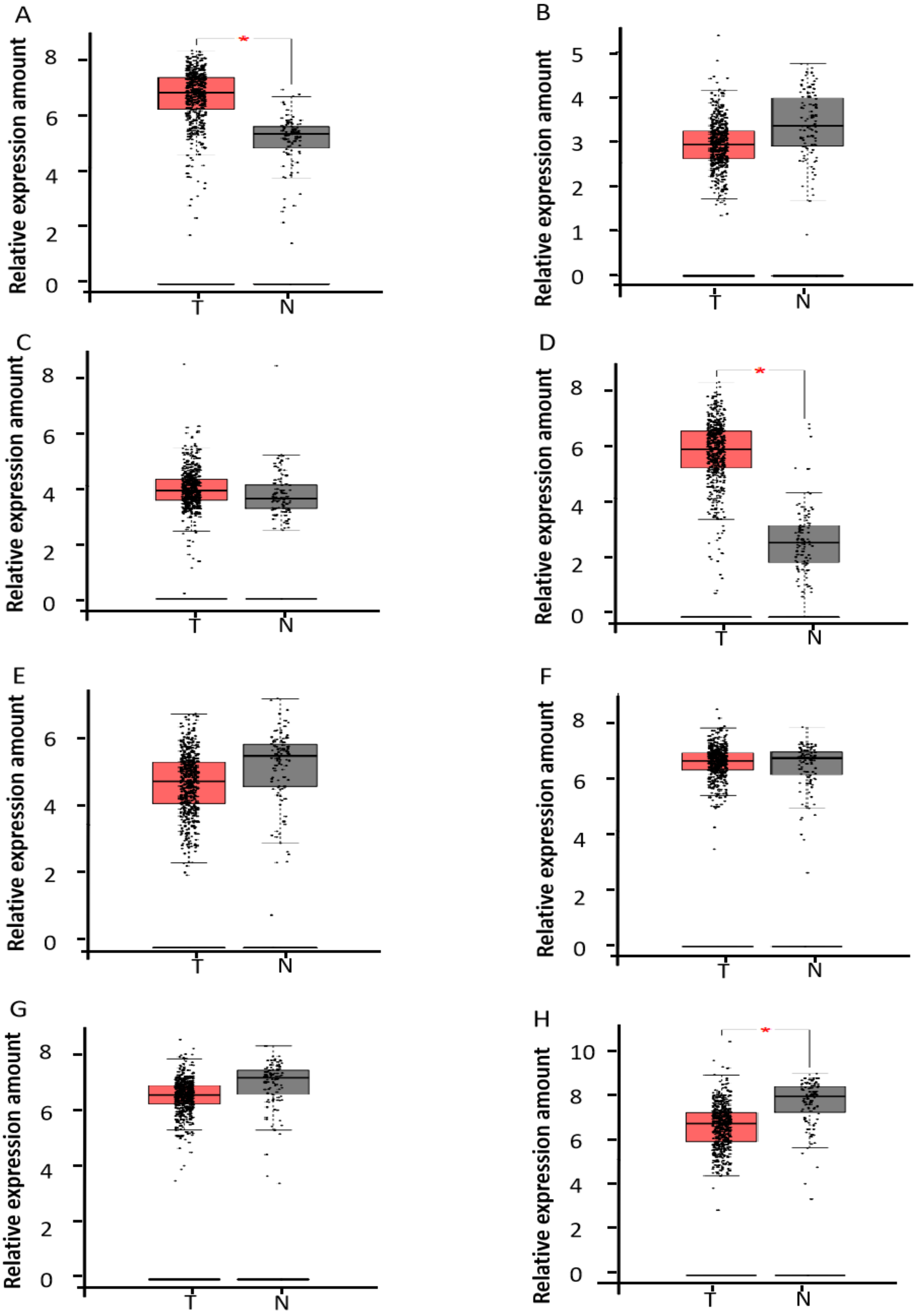

Figure S1. The box plots validated the mRNA expression profiles of FA metabolic enzymes in ccRCC and normal kidney tissues using GEPIA database. The box plot showed the relative mRNA expression of ACLY (A), ACC (B), FASN (C), SCD (D), CPT1A(E), HADHA (F), HADHB (G), and ACAT1 (H), in ccRCC (T, left, n=523) and normal tissues (N, right, n=100), respectively. * P<0.05.

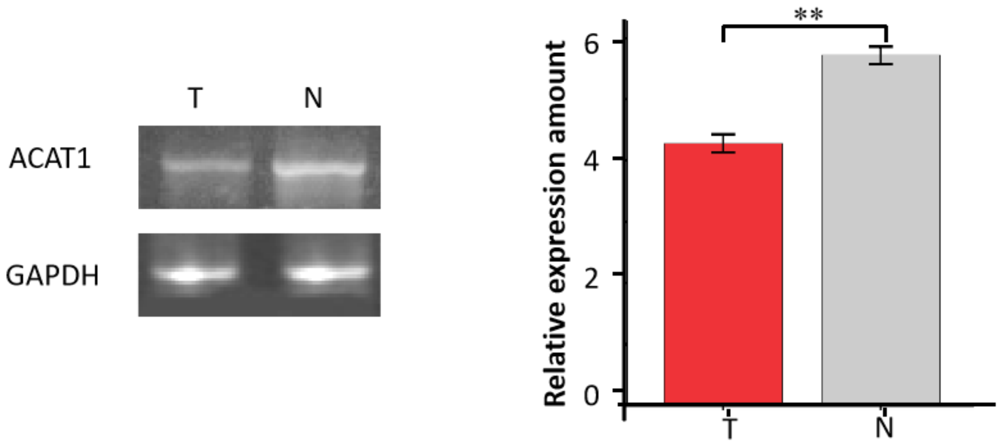

Figure S2. RT-PCR confirmed the lower mRNA expression of ACAT1 in ccRCC than normal kidney tissues. Representative ACAT1 expression by RT-PCR (left) and relative ACAT1 mRNA expression (right). T: ccRCC, N: normal tissues, n=3. * $P<0.05$, ** $P<0.01$.

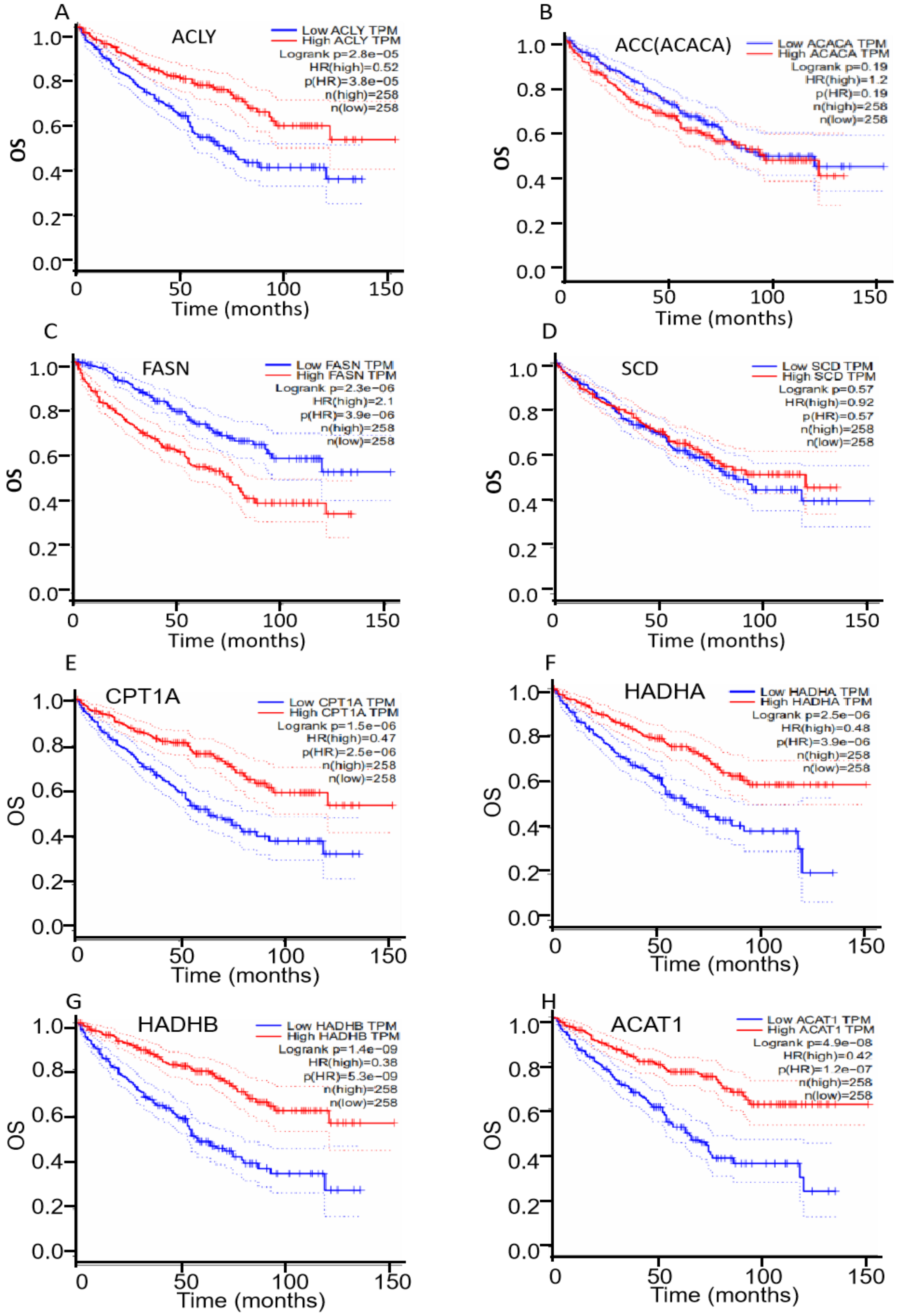

Figure S3. Overall survival (OS) curves of ACLY (A), ACC (B), FASN (C), SCD (D), CPT1A(E), HADHA (F), HADHB (G), and ACAT1 (H), were plotted by GEPIA analysis.
